# A Thyroid Genetic Classifier Correctly Predicts Benign Nodules with Indeterminate Cytology: Two Independent, Multicenter, Prospective Validation Trials

**DOI:** 10.1089/thy.2019.0490

**Published:** 2020-05-07

**Authors:** Mark Zafereo, Bryan McIver, Sergio Vargas-Salas, José Miguel Domínguez, David L. Steward, F. Christopher Holsinger, Emad Kandil, Michelle Williams, Francisco Cruz, Soledad Loyola, Antonieta Solar, Juan Carlos Roa, Augusto León, Nicolás Droppelman, Maite Lobos, Tatiana Arias, Christina S. Kong, Naifa Busaidy, Elizabeth G. Grubbs, Paul Graham, John Stewart, Alice Tang, Jiang Wang, Lisa Orloff, Marcela Henríquez, Marcela Lagos, Miren Osorio, Dina Schachter, Carmen Franco, Francisco Medina, Nelson Wohllk, René E. Diaz, Jesús Veliz, Eleonora Horvath, Hernán Tala, Pedro Pineda, Patricia Arroyo, Félix Vasquez, Eufrosina Traipe, Luis Marín, Giovanna Miranda, Elsa Bruce, Milagros Bracamonte, Natalia Mena, Hernán E. González

**Affiliations:** ^1^ Department of Head and Neck Surgery, University of Texas MD Anderson Cancer Center, Houston, Texas, USA.; ^14^ Department of Endocrine Neoplasia, University of Texas MD Anderson Cancer Center, Houston, Texas, USA.; ^15^ Department of Surgical Oncology, University of Texas MD Anderson Cancer Center, Houston, Texas, USA.; ^8^ Division of Pathology/Lab Medicine, Department of Pathology, University of Texas MD Anderson Cancer Center, Houston, Texas, USA.; ^2^ Department of Head and Neck–Endocrine Oncology, Moffitt Cancer Center, Tampa, Florida, USA.; ^3^ Department of Surgical Oncology, Pontificia Universidad Católica de Chile, Santiago, Chile.; ^4^ Department of Endocrinology, Pontificia Universidad Católica de Chile, Santiago, Chile.; ^5^ Department of Otolaryngology, Head and Neck Surgery; University of Cincinnati Medical Center, Cincinnati, Ohio, USA.; ^16^ Department of Pathology; University of Cincinnati Medical Center, Cincinnati, Ohio, USA.; ^6^ Division of Head and Neck Surgery, Department of Otolaryngology; Palo Alto, California, USA.; ^13^ Department of Pathology; Stanford University, Palo Alto, California, USA.; ^7^ Department of Surgery, School of Medicine, Tulane University, New Orleans, Louisiana, USA.; ^9^ Department of Radiology, Faculty of Medicine, Pontificia Universidad Catolica de Chile, Santiago, Chile.; ^10^ Department of Pathology, Faculty of Medicine, Pontificia Universidad Catolica de Chile, Santiago, Chile.; ^17^ Department of Laboratory Medicine, Faculty of Medicine, Pontificia Universidad Catolica de Chile, Santiago, Chile.; ^11^ Centro Diagnostico Plaza Italia, Santiago, Chile.; ^12^ Clinica UC San Carlos, Santiago, Chile.; ^18^ Clinica Santa Maria Santiago de Chile; Universidad de Chile, Santiago, Chile.; ^19^ Hospital del Salvador; Universidad de Chile, Santiago, Chile.; ^20^ Clínica Alemana de Santiago, Universidad del Desarrollo, Santiago, Chile.; ^21^ Hospital Clínico Universidad de Chile, Santiago, Chile.; ^22^ Hospital San Juan de Dios, Santiago, Chile.; ^23^ Instituto Oncológico Fundación Arturo López Pérez, Santiago, Chile.

**Keywords:** indeterminate thyroid cytology, gene classifier, clinical validation

## Abstract

***Background:*** Although most thyroid nodules with indeterminate cytology are benign, in most of the world, surgery remains as the most frequent diagnostic approach. We have previously reported a 10-gene thyroid genetic classifier, which accurately predicts benign thyroid nodules. The assay is a prototype diagnostic kit suitable for reference laboratory testing and could potentially avoid unnecessary diagnostic surgery in patients with indeterminate thyroid cytology.

***Methods:*** Classifier performance was tested in two independent, ethnically diverse, prospective multicenter trials (TGCT-1/Chile and TGCT-2/USA). A total of 4061 fine-needle aspirations were collected from 15 institutions, of which 897 (22%) were called indeterminate. The clinical site was blind to the classifier score and the clinical laboratory blind to the pathology report. A matched surgical pathology and valid classifier score was available for 270 samples.

***Results:*** Cohorts showed significant differences, including (i) clinical site patient source (academic, 43% and 97% for TGCT-1 and -2, respectively); (ii) ethnic diversity, with a greater proportion of the Hispanic population (40% vs. 3%) for TGCT-1 and a greater proportion of African American (11% vs. 0%) and Asian (10% vs. 1%) populations for TGCT-2; and (iii) tumor size (mean of 1.7 and 2.5 cm for TGCT-1 and -2, respectively). Overall, there were no differences in the histopathological profile between cohorts. Forty-one of 155 and 45 of 115 nodules were malignant (cancer prevalence of 26% and 39% for TGCT-1 and -2, respectively). The classifier predicted 37 of 41 and 41 of 45 malignant nodules, yielding a sensitivity of 90% [95% confidence interval; CI 77–97] and 91% [95% CI 79–98] for TGCT-1 and -2, respectively. One hundred one of 114 and 61 of 70 nodules were correctly predicted as benign, yielding a specificity of 89% [95% CI 82–94] and 87% [95% CI 77–94], respectively. The negative predictive values for TGCT-1 and TGCT-2 were 96% and 94%, respectively, whereas the positive predictive values were 74% and 82%, respectively. The overall accuracy for both cohorts was 89%.

***Conclusions:*** Clinical validation of the classifier demonstrates equivalent performance in two independent and ethnically diverse cohorts, accurately predicting benign thyroid nodules that can undergo surveillance as an alternative to diagnostic surgery.

## Introduction

The prevalence of thyroid nodules in the adult population reaches up to 65% (1). Expanded access to high-resolution ultrasound has significantly increased the identification of thyroid nodules and the number of fine-needle aspiration biopsies performed (2). A current limitation of cytological evaluation of fine-needle aspiration biopsies is that ∼20–25% are reported as indeterminate (3,4). Since these patients have a 15–25% risk of malignancy (3,4), they represent a significant challenge for clinical management.

Current guidelines suggest that molecular testing may be used to supplement malignancy risk assessment in lieu of proceeding directly with a strategy of either surveillance or diagnostic surgery (5). The emergence of precision medicine has provided new options intended to predict the risk of malignancy of thyroid nodules with indeterminate cytology (6,7). Currently, molecular tests are based on two approaches. One that rules in malignancy based on detection of specific DNA mutations and/or chromosomal rearrangements (8) has demonstrated high specificity and positive predictive value (PPV) to identify patients who would benefit from surgery (5). The second rules out malignancy based on genomic sequencing analysis, where a high negative predictive value (NPV) makes it possible to safely recommend surveillance (9). Tests able to rule in and rule out malignancy have been described. Such tests can reduce the need for surgery in low-risk patients while providing guidance for surgery in high-risk cases (10–12).

Currently, most of the molecular testing for indeterminate thyroid cytology is offered in the United States through centralized laboratories, which have in-house laboratory-developed tests. This significantly limits the access to molecular testing in the rest of the world where, in the absence of a diagnostic kit for local reference testing, surgery remains the most frequent choice for thyroid nodules with indeterminate cytology. We have recently reported the development of a 10-gene thyroid genetic classifier that accurately predicts benign thyroid nodules with an NPV of 96% and specificity of 87% and could potentially avoid more than 80% of unnecessary surgeries (13). The assay is built into a multiplexed quantitative polymerase chain reaction (qPCR) diagnostic kit format with a level of technical complexity that is suitable for reference laboratory testing (13). Clearance of a distributable kit by the Food and Drug Administration requires several stages of validation, including analytical and clinical studies to build an appropriate dossier for regulatory approval. In this study, we present the results of two independent, international, prospective, multicenter validation trials demonstrating a robust and consistent performance of the classifier across an ethnically diverse population.

## Methods

### Study population and protocol

**FIG. 1.**
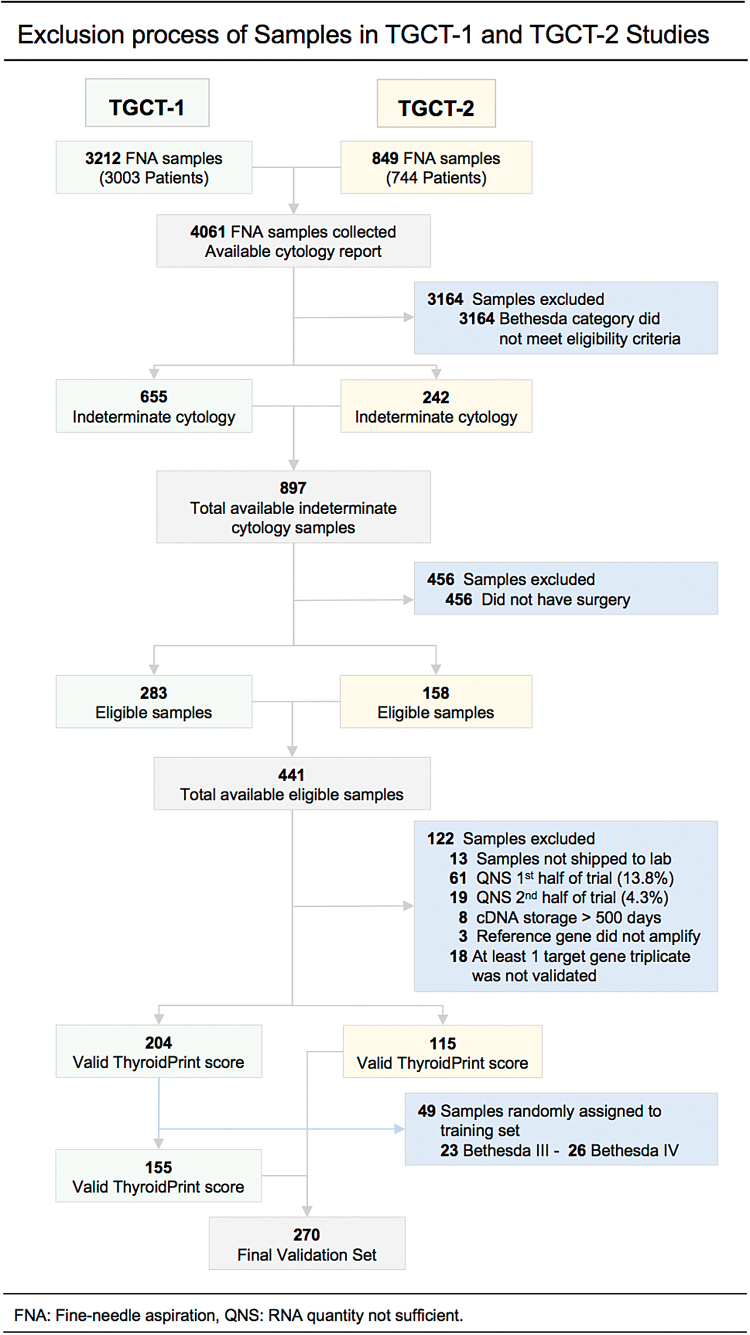
Exclusion process flowchart. Color images are available online.

Patients undergoing a fine-needle aspiration biopsy for a thyroid nodule at 15 sites from Chile and the United States were enrolled in two independent, prospective multicenter trials (Clinicaltrials.gov. TGCT-1/Chile-NCT03061318 and TGCT-2/USA-NTC03309631). Protocols were approved by local institutional ethics committees and enrolled participants provided written informed consent. Eligible patients (>18 years old with a thyroid nodule size of 10 mm or more) undergoing fine-needle aspiration were recruited from both community and academic centers. At the time of the procedure, two additional needle passes were collected and placed in RNAprotect Cell Reagent (Qiagen, Hilden, Germany). Samples were transported to the laboratory in a temperature-controlled system. Indeterminate samples underwent RNA extraction, followed by cDNA synthesis, and were stored at −20°C (Supplementary Data). Data collected included demographics and ultrasound thyroid nodule characteristics (most importantly location and size). Cytology was reported according to the Bethesda System for Reporting Thyroid Cytopathology (3). For each trial, the surgical pathology report was provided by a central expert pathologist (J.C.R. and M.W.) review. Results of the classifier were not communicated to the patient, pathologist, or treating physician. For final analysis, surgical pathology reports of malignant, noninvasive, follicular thyroid neoplasm with papillary-like nuclear features (NIFTP) (14) and follicular or Hürthle lesion of undetermined malignant potential were considered as requiring surgical management. The deidentified pathology reports and classifier scores were independently uploaded to an electronic capture system to keep the clinical site blind to the classifier score and the clinical laboratory blind to the pathology report through a password-protected system. Pathology reports were matched to the corresponding classifier result by an independent third party. Sequential sample exclusion steps are shown in Figure 1.

### Gene expression analysis

Normalization and preprocessing of qPCR data are described in the Supplementary Data. The locked classifier algorithm was derived from a training set comprising cases from Chile (169 samples), including the pooled cohort from our previous discovery study (13) (120 samples), and 49 randomly selected patients from the TGCT-1 trial (Supplementary Data). Scores from the remaining TGCT-1 set and all TGCT-2 samples were generated using the previously locked 10-gene classifier.

### Statistical analyses

Sensitivity, specificity, and area under the curve were estimated by receiver operating characteristic curves. PPVs and NPVs were estimated by Bayes' theorem. Multiple comparison tests were performed using Tukey's range test. Differences in proportions were evaluated using the *z*-test, while continuous variables were assessed for differences by the Mann–Whitney *U* test, and multiple comparisons were performed using the Kruskal–Wallis test with Dunn's *post hoc* correction. Two-sided *p*-values of less than 0.05 were considered to indicate statistical significance. Confidence intervals (CIs) are reported as two-sided 95%. All statistical analyses were performed using the SPSS, v15.0, software (SPSS, Inc., Chicago, IL), and plotting was performed using GraphPad, v7.0 (GraphPad Software, Inc., San Diego, CA).

## Results

### Characteristics of patients, thyroid nodules, and gene expression profiles

The basic demographic and thyroid nodule characteristics are shown in Table 1. The clinical performance of the classifier was validated by prospectively collecting 4061 fine-needle aspirations, of which 897 (22.0%) were called indeterminate (455 Bethesda III and 442 Bethesda IV) (Fig. 1). The final validation cohort of 270 cases was not statistically different from the 897 indeterminate cases initially enrolled—in age, sex, and thyroid nodule size (Supplementary Table S2). Furthermore, the composition of the surgical pathology of the final validation set was not significantly changed by the different stages of patient exclusion (Supplementary Table S3). The final validation set included 66% of cases from academic centers and 34% from community centers (Table 1). Although, both cohorts had a high proportion of white subjects (>50%), TGCT-1 had a higher proportion of Hispanics (40% vs. 3%), while TGCT-2 had a higher proportion of African American (11% vs. 0%) and Asian (10% vs. 1%) subjects (Table 1).

**Table 1.** Demographic and Clinical Characteristics of Study Cohorts

Variable	Cohorts					
	TGCT-1		TGCT-2		Total	
Total						
Patients	155		115		270	
FNAs	155		115		270	
Sites						
Academic	67	43%	111	97%^*^	178	66%
Community	88	57%	4	3%^*^	92	34%
Age, years						
Mean	49.4		51.8		50.2	
Range	19–80		20–85		18–85	
Sex						
Male	19	12%	20	21%	39	14%
Female	136	88%	95	79%	231	86%
Race/ethnicity						
White	79	51%	84	73%^*^	163	60%
African American	0	0%	13	11%^*^	13	5%
Hispanic	62	40%	4	3%^*^	66	24%
Asian	1	1%	11	10%^*^	12	4%
Other	13	8%	3	3%	16	6%
Nodules						
Median size (cm)	1.7		2.5		2.1	
Range size (cm)	1.0–6.1		1.0–8.5		1.0–8.5	
1.0–1.99	93	60%	42	37%^*^	135	50%
2.0–2.99	34	22%	32	28%	66	24%
3.0–3.99	16	10%	15	13%	31	11%
≥4.0	12	8%	26	23%^*^	38	14%

^*^ *p* < 0.05 TGCT-1 versus TGCT-2.

After a maximum follow-up of 4 months, a total of 441 patients underwent surgical resection (189 Bethesda III and 252 Bethesda IV) (Fig. 1). Of the 441 cases with an available surgical pathology report, 102 samples did not pass the preanalytical (80) and analytical (22) quality control criteria (Fig. 1). In the first half of the trial, 86.4% of FNA samples had sufficient RNA for testing, which improved up to 95.7% the sample RNA yield in the second half of the study.

A successful qPCR informative valid classifier score was achieved in 319 (204 cases in TGCT-1 and 115 cases in TGCT-2) of 340 (94%) samples that passed preanalytical quality control (Fig. 1). Before final analysis, 49 samples (23%) from the TGCT-1 cohort were randomly assigned to the training set (Fig. 1). The demographics, nodule sizes, and Bethesda diagnoses of assigned samples were not different from the validation set (Fig. 1 and Supplementary Table S2). The final validation set comprised 270 cases (155/TGCT-1 and 115/TGCT-2) (Fig. 1).

Comparison of the differential expression for the 10 genes (*CXCR3*, *CCR3*, *CXCL10*, *KRT19*, *TIMP1*, *CLDN1*, *CXADR*, *XMOX130*, *AFAP1L2*, and *CCR7*) (13) between surgically treated tumors and nonsurgical lesions showed that the signature followed a similar expression profile for both cohorts (Supplementary Fig. S1).

### Performance of the thyroid genetic classifier

To assess classifier performance, the surgical pathologic diagnoses were grouped to align with clinical management as nonsurgical (benign) or surgical (malignant, NIFTP, and follicular or Hürthle lesion of undetermined malignant potential) entities based on the final pathologic diagnosis and central review (Table 3). Classifier analysis of the expression of 10 genes provided a score for each sample that was categorized as benign or suspicious for malignancy.

**FIG. 2.**
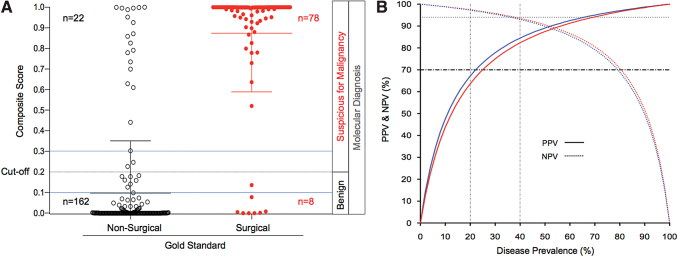
Classifier dot plot and Bayes' theorem predicted values. The thyroid genetic classifier effectively classifies indeterminate, fine-needle aspiration biopsy samples. **(A)** Dot plot of classifier scores for nonsurgical (black circles) and surgical (red circles) gold standard diagnosis are shown. Cutoff score to classify samples as nonsurgical or surgical was 0.2. Blue lines indicate a 10% range of the cutoff score. **(B)** Bayes' theorem PPVs and NPVs are shown for TGCT-1 (blue) and TGCT-2 (red). The dark horizontal dashed line is set at 70% to represent the lower limit for the PPV, and the light horizontal dotted line is set at 94% to represent the lower limit for the NPV. NPV, negative predictive value; PPV, positive predictive value. Color images are available online.

A summary of the classifier performance in the 270 matched samples (classifier score/surgical pathology) is shown in Table 2. The classifier predicted 37 of 41 and 41 of 45 surgical samples (sensitivity of 90% [95% CI 77–97] and 91% [95% CI 79–98], respectively) and 101 of 114 and 61 of 70 nonsurgical samples (specificity of 89% CI, 82–94, and 87% CI, 77–94, respectively) for the TGCT-1 and TGCT-2 cohorts, respectively. Overall, the benign call rate for both cohorts was 63%. In the pooled subset of samples reported as Bethesda III, the sensitivity was 91% [95% CI 66–100] and specificity was 92% [95% CI 71–94]. For samples reported as Bethesda IV, the sensitivity was 91% [95% CI 79–97) and specificity was 85% [95% CI 76–91]. Cancer prevalence for Bethesda subcategories III and IV and across all samples was 28%, 35%, and 32%, respectively, yielding NPVs of 96%, 94%, and 95% and PPVs of 81%, 76%, and 78%, respectively. The classifier incorrectly called 22 false positive cases, of which 14 were benign follicular nodules (9 follicular hyperplasia and 5 colloid nodules), 5 follicular adenomas, 2 Hürthle cell adenomas, and 1 case of chronic thyroiditis (Table 3). The test correctly predicted a representative spectrum of surgical histopathology subtypes commonly seen in indeterminate cytology, including papillary thyroid cancers (usual type and follicular variants). Other lesions that were correctly predicted to be surgical included follicular thyroid and Hürthle cell carcinoma, metastatic renal carcinoma, NIFTP, and follicular/Hürthle lesions of undetermined malignant potential. Due to the absence of medullary thyroid cancers (MTC) in the final validation sets, the performance of the classifier was evaluated in a separate set of FNA samples reported as MTC that were collected in the TGCT-1 trial, where the classifier predicted 100% of cases as surgical (Supplementary Table S4). False negative cases included 5 papillary thyroid carcinomas (2 conventional type and 3 follicular variant—encapsulated), none of which had aggressive features (lymph node metastasis or extrathyroidal extension). Other false negatives included 2 follicular carcinomas and 1 Hürthle cell carcinoma, all of which were minimally invasive (Supplementary Table S5). A dot plot of individual scores shows that 8 of 162 (5%) correctly classified nonsurgical and 0 of 78 (0%) of correctly classified surgical cases had a score value within the 10% range of the cutoff score (0.1–0.3) where the highest risk of misclassification occurs (Fig. 2A). Bayes' theorem analysis showed that within a disease prevalence of 20–40%, the classifier showed a minimum PPV and NPV of 70% and 94%, respectively (Fig. 2B).

**Table 2.** Performance of Thyroid Genetic Classifier

TGCT-1, Bethesda III and IV (*n* = 155, disease prevalence 26%)			
Result	Surgical (41)	Nonsurgical (114)	Test performance, % [95% CI]
Suspicious	37	13	Sensitivity, 90 (77–97)
			Specificity, 89 (82–94)
Benign	4	101	NPV, 96 (91–98)
			PPV, 74 (63–83)
			Accuracy, 89 (83–93)

TGCT-2, Bethesda III and IV (*n* = 115, disease prevalence 39%)			
Result	Surgical (45)	Nonsurgical (70)	Test performance, % [95% CI]
Suspicious	41	9	Sensitivity, 91 (79–98)
			Specificity, 87 (77–94)
Benign	4	61	NPV, 94 (86–98)
			PPV, 82 (71–89)
			Accuracy, 91 (75–94)

Bethesda III—TGCT-1 and -2 (*n* = 117, disease prevalence 28%)			
Result	Surgical (33)	Nonsurgical (84)	Test performance, % [95% CI]
Suspicious	30	7	Sensitivity, 91 (66–100)
			Specificity, 92 (71–94)
Benign	3	77	NPV, 96 (87–100)
			PPV, 81 (68–90)
			Accuracy, 91 (85–96)

Bethesda IV—TGCT-1 and -2 (*n* = 153, disease prevalence 35%)			
Result	Surgical (53)	Nonsurgical (100)	Test performance, % [95% CI]
Suspicious	5	15	Sensitivity, 91 (79–97)
			Specificity, 85 (76–91)
Benign	48	85	NPV, 94 (88–98)
			PPV, 76 (67–84)
			Accuracy, 87 (80–92)

Performance across both cohorts (*n* = 270, disease prevalence 32%)			
Result	Surgical (86)	Nonsurgical (184)	Test performance, % [95% CI]
Suspicious	78	22	Sensitivity, 91 (82–96)
			Specificity, 88 (82–92)
Benign	8	162	NPV, 95 (91–98)
			PPV, 78 (70–84)
			Accuracy, 89 (85–92)

CI, confidence interval; NPV, negative predictive value; PPV, positive predictive value.

**Table 3.** Performance Across Histopathological Subtypes

Histopathology subtype	Nodules	%	Classification benign/suspicious
Total cohort	270	100	
Nonsurgical	184	68	162/22
Benign			
Benign follicular nodule	99	54	85/14
Follicular adenoma	60	33	55/5
Follicular adenoma—Hürthle cell	10	5	8/2
Chronic lymphocytic thyroiditis	13	7	12/1
Other benign	2	1	2/0
Surgical	86	32	8/78
Malignant			
Papillary thyroid carcinoma			
Conventional variant	28	33	2/26
Follicular variant	25	29	3/22
Follicular carcinoma	14	16	2/14
Hürthle cell carcinoma	7	8	1/6
Metastatic renal cell carcinoma (clear cell)	1	1	0/1
Other			
Follicular or Hürthle cell lesion^a^ of undetermined malignant potential	3	3	0/3
NIFTP	8	9	0/8

Surgical includes surgical pathology reports of malignant, NIFTP, and follicular or Hürthle lesion of undetermined malignant potential.

^a^ Includes 2 follicular lesions and 1 Hürthle cell lesion of undetermined malignant potential.

NIFTP, noninvasive follicular thyroid neoplasm with papillary-like nuclear features.

## Discussion

This study reports the prospective clinical validation of a previously described thyroid genetic classifier (13). In two large, independent multicenter trials, we show that the classifier predicts benign thyroid nodules with an NPV of 95% and can identify true negative cases with an 88% specificity in the intended use population. Our data show strong evidence that the classifier provides the NPV needed to safely inform the benign nature of an indeterminate nodule while identifying 88% of avoidable surgeries for histologically benign cases. A key question addressed in this study is the generalizability of the classifier given the inherent risk of genetic heterogeneity between ethnically diverse populations. In this study, we show equivalent performance of the classifier in two independent cohorts with different ethnic population composition. Furthermore, for both cohorts, the differential gene expression profiles follow the same pattern, providing evidence of the robustness of the signature (Supplementary Fig. S1). The robust performance of the classifier is also shown by a dot plot where composite scores are effectively separated and a very low percent of cases fall close to the range of the cutoff score, reducing the risk of analytical uncertainty. Furthermore, despite the significant difference of disease prevalence between cohorts (26% TGCT-1 and 39% TGCT-2; Table 2), the NPV remained in the safety limit of 94% in the higher range of disease prevalence and the PPV remained above 70% in the lower limit of disease prevalence (Table 2 and Fig. 2B).

The diagnostic performance of the classifier showed high accuracy in a broad spectrum of cases that require surgical management, including papillary thyroid carcinomas (conventional and follicular variants), follicular carcinomas, Hürthle cell carcinomas, and NIFTP. Accurately predicting Hürthle cell lesions has been a challenge for molecular testing. The classifier predicted 7 of 8 (sensitivity of 88%) surgical Hürthle cell lesions (6 carcinomas and 1 undetermined malignant potential) and 8 of 10 (specificity of 80%) nonsurgical Hürthle cell lesions. Although the percent of Hürthle cell carcinomas in this study was relative low (8%), the performance in this tumor subtype is comparable with the Afirma genomic sequencing classifier and ThyroSeq v3 assays, which have reported a sensitivity of 89% and 100% for surgical lesions and a specificity of 59% and 62% for nonsurgical lesions, respectively (9,12). A limitation of this study is the absence of MTC, limiting the conclusions that can be drawn with respect to this subtype of tumors in the indeterminate setting. However, analysis in four nonindeterminate fine-needle aspiration samples showed the ability of the classifier to predict 100% MTC, providing indirect evidence of its ability to capture these tumors (Supplementary Table S4). The classifier does not have specific biomarkers for Hürthle cell lesions or MTC given that the classifier was designed to predict benign rather than malignant histology. In fact, the algorithm identifies the interactions in the expression between inflammatory and epithelial genes composing the signature to generate a robust benign profile, avoiding the need to depend on a complex and heterogeneous malignant gene expression profile (13).

False negative cases in this study did not have worrisome histopathological features, were pathologically low-risk tumors according to the American Thyroid Association, and followed a similar pattern of false negatives reported by the Afirma and ThyroSeq v3 tests (9,12). Therefore, from a clinical perspective, it can be presumed that the overall risk to patients associated with false negative diagnoses is low, more so considering that patients with thyroid nodules will continue to require a follow-up schedule based on current guidelines and recommendations (5).

This study has several strengths. First, a meaningful proportion of samples were collected from both academic (66%) and community (34%) centers, reducing potential selection bias associated with tertiary academic centers. Second, 8 of 15 sites enrolled more than 10% of cases, where all, except 1, showed a disease prevalence ranging between 18% and 43%, indicating an appropriate representation of the intended use population (Supplementary Table S6). Third, the final validation set did not show differences in age, sex, and tumor size with the initial indeterminate cohort (intent to diagnose) that could have introduced selection bias (Supplementary Table S2). In this trial, the cytology reports did not undergo a centralized review since in most of the world, this is not a routine practice, therefore keeping the real-world setting of the enrollment process. The variability in reporting thyroid cytopathology has been widely described (15,16), creating a challenge to validate molecular testing (17,18). This variability was, at least in part, addressed by the multicenter nature of this study, which systematically captures this intrinsic and unavoidable clinical reality. In addition, centralized and systematic surgical pathology reading provides evidence that the most frequent histopathology subtypes seen in indeterminate cytology were appropriately represented.

Currently, outside of the United States, there is very limited access to molecular testing due to the difficulty of overseas sample shipping and high costs of available tests. Thus, diagnostic surgery continues to be the most frequent approach for indeterminate cytology. To the best of the authors' knowledge, no clinically useful diagnostic kit has been reported to be available for indeterminate cytology. As a multianalyte algorithm assay, validation of a kit can be a challenge given rigorous controls required to guarantee reproducibility of multiple analytes and the algorithm itself, which is considered a separate medical device. For breast cancer, EndoPredict, an eight-gene qPCR classifier in a diagnostic kit format, has successfully shown robust analytical and clinical performance in its respective validation studies (19,20). In addition, EndoPredict has shown 100% reproducibility in seven laboratories, providing evidence that multianalyte qPCR gene expression diagnostic kits can be reliably deployed for local reference laboratory testing (20). The thyroid genetic classifier, which has the uniqueness that it only requires 10 genes, was designed in a multiplex qPCR diagnostic kit format such that its technical simplicity would provide a diagnostic alternative that can be potentially run on widely available qPCR diagnostic platforms used in reference laboratories. Development was performed using highly specific and sensitive TaqMan multiplexed amplification of target sequences with two reference genes, reducing the number of reactions, allowing optimized normalization of biomarker expression levels and control for an adequate qPCR. The key components of assay development are the analytical validation studies. This work is currently in progress, especially to demonstrate optimal interlaboratory reproducibility.

This study has some limitations. First, in the initial phase of patient enrollment, the sample RNA yield failure reached 14%, with failure occurring most frequently in clinical sites that did not have extensive previous experience in routine sample collection for both cytology and molecular testing. However, improved sample collection was achieved in the second half of both trials where sample RNA failure was reduced to 4% (Supplementary Table S1). Second, the rate of indeterminate cases undergoing surgery in the TGCT-2 cohort was 65%, compared with 43% in the TGCT-1 cohort, potentially introducing selection bias (Supplementary Table S1). This is likely due to the fact that most clinical sites from the TGCT-2 cohort were tertiary academic centers (97%) compared with the predominantly community center sites in the TGCT-1 cohort (57%), which was also reflected in the larger mean tumor size (Table 1) and a higher prevalence of malignancy in the TGCT-2 cohort (Table 2). However, despite these meaningful cohort differences, overall performance of the classifier proved to be similar. Third, the results presented in this study may not be fully extrapolated to alternative cytology reporting systems where the cytological classification criteria may not allow for accurately estimated predictive values that depend on the disease prevalence associated with the specific reporting system.

In conclusion, we have validated the clinical performance of a thyroid genetic classifier built into a diagnostic kit format in two independent and ethnically diverse multicenter cohorts. The technical simplicity and high accuracy of the test should provide accessible and valuable information for clinicians to identify patients who can safely undergo surveillance as an alternative to diagnostic surgery.

## Supplementary Material

Supplemental data

Supplemental data

Supplemental data

Supplemental data

Supplemental data
