# Supplementary material for: A Thyroid Genetic Classifier Correctly Predicts Benign Nodules with Indeterminate Cytology: Two Independent, Multicenter, Prospective Validation Trials
Source: Thyroid. 2020 May 7;30(5):704–12. doi: 10.1089/thy.2019.0490 (PMC7232660; doi:10.1089/thy.2019.0490)
Supplement: Supplemental data [file Supp_Data-TableS1.pdf]

Supplementary Data

Supplementary Materials and Methods

Sample collection

All samples were collected as fine-needle aspiration samples prospectively as per our protocols 14-463-CT-DS/GPDX-001 (clinicaltrials.gov NCT03061318/NTC03309631) at participating clinical sites. Samples were collected as two additional passes during the first or second aspiration pass and directly placed into RNAprotect preservative solution (Qiagen, Valencia, CA). Samples were transported chilled at 4°C and stored at −20°C upon receipt.

Cytopathology diagnoses

Following the fine needle aspiration procedure, local cytology results reported, based on the Bethesda System for Reporting Thyroid Cytopathology (S1), were collected. The local cytology report was considered indeterminate if the report was called AUS/FLUS (Bethesda III) or FN/SFN (Bethesda IV).

Surgical pathology or gold standard diagnosis

A standardized reporting nomenclature was established for surgical pathology reports (gold standard) following the 2017 World Health Organization Classification of Tumours of Endocrine Organs (S2). Detailed collection of ultrasound information, especially location in the thyroid and size, was crucial in avoiding errors in patients who had more than 1 nodule in the same thyroid lobe. When there was more than one nodule in close proximity, the surgeon would mark the index nodule that was FNA'd, such that there would be no confusion when the specimen went to pathology. Following surgery, local histopathology reports were collected. In addition, two representative slides of the index nodule were scanned in high resolution and uploaded to a secured server where, for each trial, a single expert pathologist (TGCT-1/JCR and TGCT-2/MW) blinded to the assay result provided the final pathology diagnosis. If the central expert pathologist considered that the available scanned slides were insufficient for a final diagnosis, new additional sections were scanned and uploaded. If there was a discrepancy between the expert and local reports, the expert report was considered final. No cases originally reported as malignant by local pathologists were overturned to benign, nor were benign cases overturned to malignant.

RNA isolation and cDNA synthesis

Molecular testing was performed under a strict quality system in a College of American Pathologist-accredited laboratory. Total RNA from FNAs was extracted with the RNeasy Plus Mini Kit (QIAGEN). Total RNA concentration was determined using the Qubit RNA HS Assay Kit and Qubit® 3.0 fluorometer (Invitrogen). Median RNA concentration was 8.1 ng/μL (interquartile range: 4.1–19.3). Samples with an RNA concentration ≥2 ng/μL were eligible for further analysis. Reverse transcription (RT) reactions were performed in a final volume of 20 μL by using minimum 22 ng from FNA with the ImProm-II™ Reverse Transcription System (Promega), following the manufacturer's instructions. RT reactions were performed with a minimum 22 ng of total RNA. Multiplex qPCRs were performed as previously described (S4). For sample quality control exclusions, see Supplementary Table S1.

Real-time qPCR and Cq miner data

Molecular testing was performed in a single CAP-accredited laboratory (CAP N° 1821095). All qPCRs were performed by adding 2 μL of 1:5 RT reaction dilution in a final volume of 20 μL containing 10 μL of 2×TaqMan Multiplex Master Mix with Mustang Purple (Life Technologies®, Waltham, MA), sequence-specific TaqMan assays (Thermo Fisher®, Waltham, MA), and nuclease-free water. All qPCRs were run in the Rotor-Gene Q thermocycler (Qiagen, Hilden, Germany). Positive (a mixture of benign and malignant thyroid tissue RNA) and negative (water only) controls were included in each assay run. Conditions for amplification were 10 minutes at 95°C, followed by 40 cycles of 10 seconds at 95°C, and 20 seconds at 60°C. In our initial work, raw qPCR data were represented by the cycle threshold (Ct), which requires performing a standard curve to set the threshold cutoff. It has been shown that Ct values can present variations due to preanalytical and analytical factors (reagent lots, equipment, and operators). For analytical quality control, we performed a review of raw Ct values for housekeeping genes, which showed up to 2–3 Ct value shifts across all 10-gene qPCRs between cohorts. To address this analytical variation, raw qPCR data were extracted using a cycle quantification miner algorithm (Cq) method (S3). This algorithm avoids the need to perform a new standard curve to determine the threshold for each qPCR batch and provides an objective and noise-resistant method for quantification of qPCR results, which is independent of the specific equipment used (S3). The Cq miner algorithm was coded using RStudio and Cq values were obtained from the historical discovery cohort (S4), the TGCT-1 and TGCT-2 trials. Housekeeping genes showed less than 1.0 Cq difference across all 10-gene qPCRs between cohorts and our discovery study, therefore Cq was used for the final classifier analysis. For normalization, a ratio was calculated by dividing the geometric mean of the housekeeping gene Cq values by the target gene Cq value.

The training set and validation process

The training set included a total of 169 cases, 120 samples were pooled from the discovery cohort (S4) and 49 randomly selected samples form the TGCT-1 trial. After data preprocessing, the classifier was trained by using a multilayer perceptron neural network with categorical cross-entropy for sampling mitigation bias and locked before validation cohort data analysis. The training process did not include data from validation cohorts. An independent biostatistician, not involved in the training process and blind to the surgical pathology gold standard, analyzed Cq values of the validation cohorts using the previously locked classifier to produce composite scores that were uploaded to a password-protected database. Deidentified, coded, surgical pathology gold standards were matched to respective scores by an independent third party for final analysis.

Supplementary Table S1. Study Exclusion Process

Trial	*TGCT-1*	%	*TGCT-2*	%	*Total*	%
Consented patients	3261		856		4117	100.0
Screening failure						
Age <18 years	0	0.0	0	0.0	0	0.0
Nodule <8 mm	16	0.5	0	0.0	16	0.4
Previous history of bleeding	0	0.0	0	0.0	0	0.0
Patient cannot sign IC	0	0.0	0	0.0	0	0.0
Sample not collected	0	0.0	6	0.7	6	0.1
Remaining subtotal	3245	99.5	850	99.3	4095	99.5
Eligible patients + cytology report not available	33	1.02	1	0.12	34	0.8
Remaining subtotal	3212	99.0	849	99.9	4061	99.2
Nonindeterminate Bethesda I, II, V, and VI	2557	79.6	607	71.5	3164	77.9
Bethesda III	282	8.8	173	20.4	455	11.2
Bethesda IV	373	11.6	69	8.1	442	10.9
Total indeterminate cases	655	20.4	242	28.5	897	22.1
Surgical biopsy not available by cutoff date	372	58.3	95	39.3	456	53.2
Bethesda III	85	30.1	104	60.1	189	41.5
Bethesda IV	198	53.1	54	78.3	252	57.0
Indeterminate + surgical Bp gold standard	283	43.2	158	65.3	441	49.2
Preanalytical and analytical exclusion						
Sample not shipped	7	2.5	6	3.8	13	2.9
Insufficient RNA (QNS) first half of trial	39	13.8	22	13.9	61	13.8
Insufficient RNA (QNS) second half of trial	12	4.2	7	4.4	19	4.3
cDNA storage >500 days	8	2.8	0	0.0	8	1.8
Reference gene did not amplify	2	0.7	1	0.6	3	1.1
At least one target gene triplicate was not validated	11	3.9	7	4.4	18	6.7
Indeterminate + surgical Bp gold standard + valid ThyroidPrint score	204	72.1	115	72.8	319	72.3
Randomly assigned to training set	49	24	0	0	49	
Remaining samples	155	76	115	100	270	
Valid classifier score	155	0.7598	115	1	270	

QNS, quantity not sufficient.

Supplementary References

S1. Cibas ES, Ali SZ 2009 The Bethesda System for reporting thyroid cytopathology. Thyroid **19:**1159–1165.

S2. Lloyd R, Osamura R, Klöppel G, Rosai J; World Health Organization International Agency for Research on CancerWHO Classification of Tumours of Endocrine Organs. Fourth edition. Lyon, France.

S3. Zhao S, Fernald R 2005 Comprehensive algorithm for quantitative real-time polymerase chain reaction. J Computat Biol **12:**1047–1064.

S4. González HE, Martínez JR, Vargas-Salas S, Solar A, Veliz L, Cruz F, Arias T, Loyola S, Horvath E, Tala H, Traipe E, Meneses M, Marín L, Wohllk N, Diaz RE, Véliz J, Pineda P, Arroyo P, Mena N, Bracamonte M, Miranda G, Bruce E, Urra S 2017 A 10-gene classifier for indeterminate thyroid nodules: development and multicenter accuracy study. Thyroid **27:**1058–1067.
