# Supplementary material for: A Thyroid Genetic Classifier Correctly Predicts Benign Nodules with Indeterminate Cytology: Two Independent, Multicenter, Prospective Validation Trials
Source: Thyroid. 2020 May 7;30(5):704–12. doi: 10.1089/thy.2019.0490 (PMC7232660; doi:10.1089/thy.2019.0490)
Supplement: Supplemental data [file Supp_TableS2.pdf]

SUPPLEMENTARY TABLE S2. DETAILED DEMOGRAPHIC AND CLINICAL CHARACTERISTICS OF THE STUDY COHORT

Variable	*Indeterminate*				*Indeterminate + surgery*				*Final validation set*				*Samples randomly assigned from TGCT-1 to the training set*	
Total	*TGCT-1*	*TGCT-2*	*Total*	%	*TGCT-1*	*TGCT-2*	*Total*	%	*TGCT-1*	*TGCT-2*	*Total*	%		%
Patients	616	223	839	23	276	148	424	51	153	115	268	63	49	
FNAs	655	242	897	22	283	158	441	49	155	115	270	61	49	
Sites														
Academic	316	221	537	60	116	152	268	61	67	111	178	66	22	45
Community	339	21	360	40	167	6	173	39	88	4	92	34	27	55
Age, years														
Mean	51.1	55.4	52.8		49.5	52.4	51.1		49.4	51.8	50.6		48.4	
Range	19–83	20–88	19–88		19–83	20–85	20–85		19–80	20–85	19–85		20–78	
Sex														
Male	75	55	130	14	37	29	66	15	19	20	39	14	7	14
Female	580	187	767	86	246	129	375	85	136	95	231	86	42	86
Race/ethnicity														
White	406	150	556	62	170	95	265	60	79	84	163	60	26	53
African American	20	7	27	3	14	8	22	5	0	13	13	5	0	0
Hispanic	151	56	206	23	65	36	101	23	62	4	66	24	21	43
Asian	13	5	18	2	11	6	18	4	1	11	12	4	0	0
Other	66	24	90	10	23	13	35	8	13	3	16	6	2	4
Nodules														
Median size (cm)	1.9	2.5	2.2		1.9	2.8	2.3		1.7	2.9	2.4		1.8	
Range size (cm)	1.0–8.0	1.0–8.5	1.0–8.5		1.0–8.0	1.0–8.0	1.0–8.0		1.0–6.1	1.0–8.5	1.0–8.5		1.0–5.2	
1.0–1.99 cm	376	95	471	53	155	58	213	52	109	42	151	51	27	55
2.0–2.99 cm	161	63	224	25	61	39	100	24	43	30	73	24	13	27
3.0–3.99 cm	73	38	111	12	22	25	47	11	18	17	35	12	5	10
>4.0 cm	45	46	91	10	15	36	51	12	13	26	39	13	4	8

No statistical differences were found for any demographic or nodule characteristic between all indeterminate cases enrolled and the final validation set.
