# Supplementary material for: A Thyroid Genetic Classifier Correctly Predicts Benign Nodules with Indeterminate Cytology: Two Independent, Multicenter, Prospective Validation Trials
Source: Thyroid. 2020 May 7;30(5):704–12. doi: 10.1089/thy.2019.0490 (PMC7232660; doi:10.1089/thy.2019.0490)
Supplement: Supplemental data [file Supp_FigS1-TableS3.pdf]

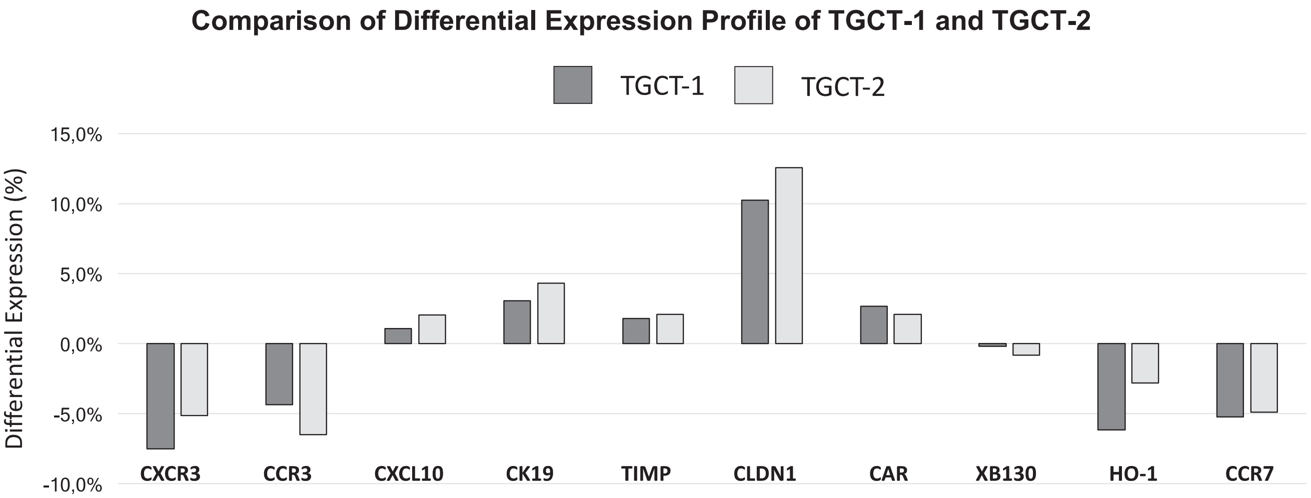

SUPPLEMENTARY FIG. S1. Comparison of differential expression profile of TGCT-1 and TGCT-2. Zero represents the mean gene expression of benign tumors. Bars represent the magnitude of differential expression of malignant tumors with respect to benign tumors.

Supplementary Table S3. Comparison of Pathology Rates After Exclusions

	Cases with surgical pathology before exclusions in TGCT-1 and -2		*Final Validation TGCT-1 and -2*	
Histopathology subtype	*Nodules*	*%*	*Nodules*	*%*
Total cohort	441		270	
Nonsurgical	315	71	184	68
Benign				
Benign follicular nodule	175	56	99	46
Follicular adenoma	98	31	60	28
Follicular adenoma—Hürthle cell	17	5	10	5
Chronic lymphocytic thyroiditis	19	6	13	6
Other benign	6	2	2	1
Surgical	126	29	86	32
Malignant				
Papillary thyroid carcinoma				
Conventional variant	46	37	29	34
Follicular variant	38	30	25	29
Follicular carcinoma	18	14	14	16
Hürthle cell carcinoma	7	6	6	7
Metastatic renal cell carcinoma (clear cell)	1	1	1	1
Others				
Follicular or Hürthle cell lesion of undetermined malignant potential	3	2	3	3
NIFTP	13	10	8	9

NIFTP, noninvasive follicular thyroid neoplasm with papillary-like nuclear features.
