# Supplementary material for: A Thyroid Genetic Classifier Correctly Predicts Benign Nodules with Indeterminate Cytology: Two Independent, Multicenter, Prospective Validation Trials
Source: Thyroid. 2020 May 7;30(5):704–12. doi: 10.1089/thy.2019.0490 (PMC7232660; doi:10.1089/thy.2019.0490)
Supplement: Supplemental data [file Supp_TableS4-S5.pdf]

Supplementary Table S4. Performance of Thyroid Genetic Classifier in Low-Frequency Tumors

Patient/trial	*Sex*	*Bethesda*	*Size (mm)*	*Surgical pathology*	*Final diagnosis*	*mRNA (μg/μL)*	*Classifier score*	*Molecular diagnosis*
1/TGCT-1	Female	VI (MTC)	25	MTC	Malignant	155.0	0.998	Malignant
2/TGCT-1	Female	VI (MTC)	9	MTC	Malignant	23.0	0.991	Malignant
3/TGCT-1	Male	VI (MTC)	12	MTC	Malignant	67.0	1000	Malignant
4/TGCT-1	Male	VI (MTC)	19	MTC	Malignant	398.0	1000	Malignant

MTC, medullary thyroid carcinoma.

Supplementary Table S5. Surgical Pathology of False Negative Cases

Trial	*Gender*	*Bethesda*	*Size (cm)*	*Definitive central pathology review*	*mRNA (μg/μL)*	*Classifier score*
TGCT-1	Female	III (AUS/FLUS)	1.4	PTC—follicular variant—encapsulated	27.7	0.000
TGCT-1	Female	IV (follicular neoplasm)	1.0	PTC—conventional type	53.8	0.078
TGCT-1	Female	IV (follicular neoplasm)	3.1	FTC—minimally invasive	6.68	0.000
TGCT-1	Female	IV (follicular neoplasm)	1.1	PTC—Follicular Variant—Encapsulated	172	0.000
TGCT-2	Female	III (AUS/FLUS)	3.9	Hurthle cell carcinoma—minimally invasive	252.0	0.008
TGCT-2	Female	IV (follicular neoplasm)	2.1	PTC—conventional type	647.0	0.001
TGCT-2	Female	IV (follicular neoplasm)	3.0	FTC—minimally invasive	4.1	0.132
TGCT-2	Female	III (AUS/FLUS)	2.6	PTC—follicular variant—encapsulated	40.4	0.004

AUS–FLUS, atypia of undetermined significance or follicular lesion of undetermined significance; FTC, follicular thyroid carcinoma; PTC, papillary thyroid carcinoma.
