# Supplementary material for: A Thyroid Genetic Classifier Correctly Predicts Benign Nodules with Indeterminate Cytology: Two Independent, Multicenter, Prospective Validation Trials
Source: Thyroid. 2020 May 7;30(5):704–12. doi: 10.1089/thy.2019.0490 (PMC7232660; doi:10.1089/thy.2019.0490)
Supplement: Supplemental data [file Supp_TableS6.pdf]

Supplementary Table S6. Detailed Description of Clinical Site Type and Patient Enrollment Per Site

Trial	*Site*	*Clinical site type*	*Nonsurgical*	*Surgical*	*Total*	*Disease prevalence (%)*	*Percent of cohort (%)*
TGCT-1	1	Community	3	1	4	25	2.6
TGCT-1	2	Community	21	6	27	22	17.4
TGCT-1	3	Academic	23	5	28	18	18.1
TGCT-1	4	Community	33	8	41	20	26.5
TGCT-1	5	Community	11	5	16	31	10.3
TGCT-1	6	Academic	1	0	1	0	0.6
TGCT-1	7	Academic	4	0	4	0	2.6
TGCT-1	8	Academic	1	0	1	0	0.6
TGCT-1	9	Academic	17	16	33	48	21.3
Subtotal TGCT-1			114	41	155	26	100
TGCT-2	10	Community	4	0	4	0	3.5
TGCT-2	11	Academic	9	0	9	0	7.8
TGCT-2	12	Academic	26	32	58	55	50.4
TGCT-2	13	Academic	4	3	7	43	6.1
TGCT-2	14	Academic	10	4	14	29	12.2
TGCT-2	15	Academic	17	6	23	26	20.0
Subtotal TGCT-2			70	45	115	39	100.0
Total validation set			184	86	270	32	100
